# Supplementary material for: Pre-Referral Primary Care Blood Tests and Symptom Presentation before Cancer Diagnosis: National Cancer Diagnosis Audit Data
Source: Cancers (Basel). 2023 Jul 12;15(14):3587. doi: 10.3390/cancers15143587 (PMC10377509; doi:10.3390/cancers15143587)
Supplement: Supplementary file 1 [file cancers-15-03587-s001.zip › cancers-2474858-SI.pdf]

cancers

Supplementary File

Table S1: Assignment of symptoms for supplementary analysis:

0.01 – 0.99% PPV (n=12)	1 – 1.99% PPV (n=13)	2 - 2.99% PPV (n=5)	PPV 3 - 4.99% (n=3)	PPV ≥5% (n=9)
Deep vein thrombosis (**NICE**)<br>Urinary tract infection (**NICE**)<br>Chest infection (**NICE**).<br>Headache (**NICE**)<br>Fracture (**NICE**)<br>Back pain (**NICE**)<br>Loss of appetite (**NICE**)<br>Bone pain (**NICE**)<br>Lymphadenopathy (generalised or localised – **NICE**)<br>Fever (**NICE**)<br>Visual disturbance or loss (**NICE**)<br>Gastrointestinal reflux (**NICE**)	Breast Pain (**Moore**)<br>Dyspnoea (**Moore**)<br>Dysuria (**Moore**)<br>Diarrhoea (**Moore**)<br>Nausea and/or vomiting (**Moore**)<br>Constipation (**Moore**)<br>Fatigue (**Moore**)<br>Dyspepsia (**Moore**)<br>Chest pain (**Moore**)<br>Cough (**Moore**)<br>vaginal discharge (**Moore**)<br>Fit / Seizure (**NICE**)<br>Upper Abdominal pain (**NICE**).	Abdominal pain (NOS) (**Moore**)<br>Weight loss (**Moore**)<br>Hoarseness (**Moore**)<br>haematemesis (**Moore**)<br>Distension (**Moore**)	Change in bowel habit (**Moore**)<br>Haematuria (**Moore)**<br>nipple changes (**Moore**)	Haemoptysis (**Moore**)<br>Dysphagia (**Moore**)<br>Jaundice (**Moore**),<br>Rectal bleeding (**Moore**)<br>Breast lump/mass (**Moore)**<br>nipple discharge (**Moore**)<br>Post-menopausal bleeding (**Moore**)<br>LUTS (nocturia, frequency, hesitancy, urgency, retention) (**Moore**)<br>Non-pigmented lesion (**NICE**)

* Symptom allocation based on **Moore** *et al* (2021) [18] and **NICE** guidelines (NG12) – Evidence available on request.
